# Preferences and beliefs of Dutch orthopaedic surgeons and patients reduce the implementation of “Choosing Wisely” recommendations in degenerative knee disease

**DOI:** 10.1007/s00167-019-05708-8

**Published:** 2019-09-25

**Authors:** T. Rietbergen, R. L. Diercks, I. Anker-van der Wel, M. E. van den Akker-van Marle, N. Lopuhaä, R. P. A. Janssen, H. M. J. van der Linden-van der Zwaag, R. G. H. H. Nelissen, P. J. Marang-van de Mheen, L. van Bodegom-Vos

**Affiliations:** 1 Department of Biomedical Data Sciences, Section Medical Decision Making, Leiden University Medical Center, Postzone J10-s, P.O. Box 9600, 2300 RC Leiden, The Netherlands; 2 Department of Orthopaedics, University Medical Center Groningen, Groningen, The Netherlands; 3 ReumaNederland, Amsterdam, The Netherlands; 4 Department of Orthopaedics, Máxima Medical Center, Eindhoven, The Netherlands; 5 Department of Orthopaedics, Leiden University Medical Center, Leiden, The Netherlands

**Keywords:** Choosing Wisely, Degenerative knee disease, Magnetic resonance imaging, Knee arthroscopy, De-implementation, Barriers and facilitators

## Abstract

**Purpose:**

The purpose of this study was to assess which factors were associated with the implementation of “Choosing Wisely” recommendations to refrain from routine MRI and arthroscopy use in degenerative knee disease.

**Methods:**

Cross-sectional surveys were sent to 123 patients (response rate 95%) and 413 orthopaedic surgeons (response rate 62%) fulfilling the inclusion criteria. Univariate and multivariate logistic regression analyses were used to identify factors associated with implementation of “Choosing Wisely” recommendations.

**Results:**

Factors reducing implementation of the MRI recommendation among patients included explanation of added value by an orthopaedic surgeon [OR 0.18 (95% CI 0.07–0.47)] and patient preference for MRI [OR 0.27 (95% CI 0.08–0.92)]. Factors reducing implementation among orthopaedic surgeons were higher valuation of own MRI experience than existing evidence [OR 0.41 (95% CI 0.19–0.88)] and higher estimated patients’ knowledge to participate in shared decision-making [OR 0.38 (95% CI 0.17–0.88)]. Factors reducing implementation of the arthroscopy recommendation among patients were orthopaedic surgeons’ preferences for an arthroscopy [OR 0.03 (95% CI 0.00–0.22)] and positive experiences with arthroscopy of friends/family [OR 0.03 (95% CI 0.00–0.39)]. Factors reducing implementation among orthopaedic surgeons were higher valuation of own arthroscopy experience than existing evidence [OR 0.17 (95% CI 0.07–0.46)] and belief in the added value [OR 0.28 (95% CI 0.10–0.81)].

**Conclusions:**

Implementation of “Choosing Wisely” recommendations in degenerative knee disease can be improved by strategies to change clinician beliefs about the added value of MRIs and arthroscopies, and by patient-directed strategies addressing patient preferences and underlying beliefs for added value of MRI and arthroscopies resulting from experiences of people in their environment.

**Level of evidence:**

IV.

## Introduction

Approximately, 25% of patients aged 50 years and over experience knee symptoms from degenerative knee disease [37, 41]. These patients suffer from pain during walking, climbing stairs and squatting, and have functional loss [15, 26]. In some cases, knee range of motion is limited due to a meniscal tear, also known as locking symptoms. These degenerative meniscal tears could be symptoms of early stage osteoarthritis [18, 19].

For diagnosing patients with degenerative knee disease, clinical practice guidelines [2, 4, 7, 8] and literature recommend weight-bearing radiographs (fixed flexion view—Rosenberg view) to determine the presence and severity of degenerative knee disease and to exclude other causes of knee pain, such as osteonecrosis of the femoral condyle or tibial plateau [18, 45]. Although MRI has high sensitivity and specificity in detecting meniscal tears in older patients [18, 39], routine use of MRI is not recommended for diagnosis because of the poor correlation with patient symptoms [14, 19, 20, 33]. Similarly, clinical practice guidelines do not recommend the use of arthroscopic surgery as there is no benefit shown of arthroscopic surgery over non-surgical treatments such as exercise therapy, analgesic medication and dietary advice [2, 17, 18, 27, 29–31, 37, 38, 43, 44]. If locking symptoms are present, or if pain is not reduced after non-surgical treatments, arthroscopy may be warranted. So, MRI and arthroscopic surgery without prior conservative management in degenerative knee disease can be considered as unnecessary or low value care as these provide no benefit for the patient, waste resources and may even cause harm to the patient [17, 35].

Although practice guidelines and the underlying evidence do not recommend routine use of MRI and arthroscopy, many patients aged 50 years and over with degenerative knee disease receive an MRI and/or a knee arthroscopy [9, 13, 16, 17, 24, 28, 32, 40]. Arthroscopic knee surgery is even the most common orthopaedic procedure in countries with available data and is, on a global scale, performed more than two million times each year [37].

In an effort to reduce the unnecessary use of MRIs and knee arthroscopies for patients with degenerative knee disease, medical societies in several countries have formulated “Choosing Wisely” recommendations regarding their use [1, 3, 6, 10]. A recent study of Rosenberg et al. [34] showed that developing such recommendations does not necessarily eradicate low value care. To stimulate the implementation of the CW recommendations, interventions should be adapted to the factors associated with implementation of specific CW recommendations—in this case ‘do not order an MRI for suspected degenerative meniscal tears’ and ‘do not perform knee arthroscopy for patients with degenerative meniscal tears of degenerative knee disease without mechanical symptoms’ [42]. Previous research has suggested that conducting knee arthroscopies is driven by clinician beliefs in the effectiveness [24, 28], the need to meet patient expectations [12], perverse financial incentives for clinicians/hospitals [24, 28], fragmented clinical decision pathways [24], and insurance coverage [32]. However, no study has systematically studied factors influencing the implementation of these CW recommendations on degenerative knee complaints in patients of 50 years and older.

Therefore, the aim of this study is to investigate which factors are associated with implementation of CW recommendations among patients and orthopaedic surgeons in the Netherlands which aim to reduce the number of unnecessary MRIs and arthroscopies in patients aged 50 years and over with degenerative knee disease. Based on the previous research above, it was hypothesized that orthopaedic surgeons’ beliefs in the effectiveness of MRI and knee arthroscopy, the need to meet patient expectations, perverse financial incentives and insurance coverage all hamper the implementation of CW recommendations.

## Materials and methods

To investigate which factors are associated with implementation of CW recommendations, cross-sectional online surveys were performed among Dutch patients ≥ 50 years with degenerative knee disease and orthopaedic surgeons specialized in knee pathology (members of Dutch Knee Society) throughout the Netherlands. In the Netherlands, patients with (suspected) degenerative knee disease first visit a general practitioner before being referred to an orthopaedic surgeon.

A literature search and semi-structured interviews among Dutch patients with degenerative knee disease (*N* = 3) and orthopaedic surgeons (*N* = 3) were performed to identify potential factors influencing implementation of CW recommendations regarding MRIs and arthroscopies in patients ≥ 50 years with degenerative knee disease. For the interviews, purposive sampling was applied to obtain contrasting views, thereby identifying a broad spectrum of potential factors. Patients ≥ 50 years with degenerative knee problems who did and did not have an MRI and/or arthroscopy, and orthopaedic surgeons who either do or do not perform an MRI and/or arthroscopy in these patients were selected. The interview questions were based on the framework of Grol and Wensing [23]. This framework distinguishes factors influencing implementation at the following six levels: (a) innovation, (b) individual professional, (c) patient, (d) social context, (e) organisational context as well as the (f) economic and political context.

The semi-structured interviews were audio-taped, fully transcribed and analysed using open coding. The qualitative analysis was performed using the software program ATLAS.ti (version 7.5.16). A total of 55 factors were identified from the literature [21, 22, 25, 36, 46] for orthopaedic surgeons and patients. Besides, four factors were added based on the interviews among orthopaedic surgeons and patients. Overall, 59 factors were found, 26 for the patient and 33 for the orthopaedic surgeon.

### Survey for patients

The survey included items about (1) background characteristics, (2) characteristics of the received care and (3) factors influencing implementation of the CW recommendations regarding MRI and arthroscopy. The items of these first two categories are given in “Appendix 1: Items survey patient”. The third part of the survey about factors influencing implementation of the CW recommendations consisted of 26 items identified in the interviews and literature. Answers could be given on a 4-point Likert scale, ranging from “totally agree” (coded 1) to “totally disagree” (coded 4) and some questions could be answered with yes/no. If the patient underwent an MRI or arthroscopy, additional questions followed, for example on waiting time.

#### Population

Patients were recruited via advertisements in newspapers and on websites of patient organisations. Assuming a baseline implementation rate of 15% in those with a certain barrier for implementation, sample size calculations showed that at least 120 patients would be needed to be able to detect a twofold increase odds in those without the barrier with 80% power and 95% reliability. The developed survey was sent to a sample of patients with degenerative knee disease (*N* = 138). Inclusion criteria were: age ≥ 50 years; degenerative knee disease; consultation with an orthopaedic surgeon for their degenerative knee disease. Patients on a waiting list for a total knee arthroplasty (TKA) or who already received a TKA were excluded. Also, patients with an inability to understand written Dutch were excluded. If patients indicated that they preferred to fill in the survey on paper rather than online, they received a paper survey. Two reminders were sent in case of non-response, one after 6 and one 12 weeks after the initial invitation. Patients received a ten euro gift card as an incentive upon completion of the survey.

### Survey for orthopaedic surgeons

The survey for orthopaedic surgeons included items regarding (1) background characteristics, (2) characteristics of care delivery and (3) factors influencing implementation of the CW recommendations. The items of these first two categories are given in “Appendix 2: Items survey orthopaedic surgeon”. The third part consisted of 33 items covering the factors influencing implementation of the CW recommendations for orthopaedic surgeons. Answers could be given on a 4-point Likert scale, ranging from “totally agree” (coded 1) to “totally disagree” (coded 4).

#### Population

All Dutch orthopaedic surgeons specialized in knee pathology listed with an email address in the registry of the Dutch Orthopaedic Association (NOV) were invited by email to participate in the current study (*N* = 422). Inclusion criterion was: treatment of patients ≥ 50 years with degenerative knee symptoms. This criterion was asked as the first question of the survey. Non-responders received two reminders, one after 2 weeks and another 4 weeks after the initial invitation.

The Medical Ethical Committee (CME P16.190/NV/nv) of the Leiden University Medical Center confirmed that ethical approval for this type of study was not required under Dutch law.

### Statistical analysis

Data from all respondents who completed the survey and fulfilled the inclusion criteria were included in the analyses. Descriptive statistics were used to describe the background characteristics, the care received by the patients, and characteristics of the care delivery according to the orthopaedic surgeon. The factors influencing implementation were dichotomized into agree ‘1’ (totally agree and agree) and disagree ‘0’ (totally disagree and disagree), because of few observations in some categories of the original Likert scale. If patients had an MRI and/or an arthroscopy, implemented CW recommendation was coded as 0 (no) and as 1 (yes) otherwise.

For patients, univariate logistic regression analysis was first used to assess which background characteristics, received care and potential factor for implementation were associated with the implemented CW recommendation, with MRI and arthroscopy (‘1’ yes and ‘0’ no) as the dependent variable. A similar analysis was conducted for orthopaedic surgeons, with self-reported implementation of the MRI/arthroscopy recommendations (yes/no) as dependent variable and background characteristics, care delivery characteristics and the factors influencing implementation of the CW recommendations (agree/disagree) as independent variables.

In addition, for both patients and orthopaedic surgeons, a multivariate logistic regression analysis was performed including those background characteristics, characteristics of the received care/care delivery and the factors influencing the CW recommendations with a *p* value ≤ 0.10 in univariate analyses. All analyses were performed using the software package SPSS (IBM SPSS, version 23).

## Results

Of the 138 recruited patients, 131 completed the survey (response rate 95%). Fifteen were excluded because they did not fulfil the inclusion criteria (“Appendix 3: Flowcharts”). Of the 422 invited orthopaedic surgeons, 261 completed the survey (response rate 62%). Nine were excluded because they did not treat any patients ≥ 50 years with degenerative knee disease. Table 1 shows that the majority of the patients were female (61%) receiving higher education (47%), with average age 63.2 years. The majority of patients had additional coverage in their insurance (85%). In the Netherlands, patients are obliged to have a basic insurance with or without an additional coverage. The basic insurance has a mandatory excess of 385 euro. Patients who completed the survey represented the target group well, compared to the characteristics of Dutch orthopaedic patients [5]. Most of the orthopaedic surgeons who responded were male (90%), with an average age of 47.2 years and 12.0 years of working experience (Table 2). This was a realistic representation of the orthopaedic workforce in the Netherlands. The largest group worked in a general hospital (41%) in the middle region of the Netherlands (42%). Most of these orthopaedic surgeons saw more than 20 new patients per month (78%).

**Table 1:** Background characteristics of patients and received care from a patient perspective (*n *= 116)

Background characteristics	
Age in years, mean (SD)	63.2 (7.9)
Female, *n* (%)	71 (61.2)
Region of residence, *n* (%)	
North	38 (32.8)
Middle	68 (58.6)
South	10 (8.6)
Education, *n* (%)	
Basic	8 (6.9)
Intermediate	53 (45.7)
High	55 (47.4)
Start of symptoms of degenerative knee disease, *n* (%)	
≤ 1 year ago	18 (15.4)
> 1 year ago	98 (84.5)
Diagnosis of locking symptoms by orthopaedic surgeon^e^, *n* (%)	7 (12.5)
Pain before consult with orthopaedic surgeon (VAS), mean (SD)^f^	7.1 (2.2)
Pain at this moment (VAS), mean (SD)^f^	4.7 (2.2)
Type of insurance, *n* (%)	
Basic only	17 (14.7)
Basic with additional coverage	99 (85.3)
Received care	
Patient visited …, *n* (%)	
General practitioner (GP)	103 (88.8)
Physical therapist	85 (73.3)
Dietician	10 (8.6)
Other primary care specialists	13 (11.2)
Patient underwent …, *n* (%)	
MRI scan	74 (63.8)
Arthroscopy	56 (48.3)
Time between the start of knee complaints and the consultation with the general practitioner, *n* (%)^c^	
≤ 6 weeks	47 (51.1)
> 6 weeks	45 (48.9)
Time between consultation with the general practitioner and orthopaedic surgeon, *n* (%)^d^	
≤ 6 weeks	83 (80.6)
> 6 weeks	20 (19.4)
Waiting time for MRI scan^b^, *n* (%)	
≤ 2 weeks	40 (66.7)
> 2 weeks	20 (33.3)
Waiting time for arthroscopy^a^	
≤ 2 weeks	11 (23.9)
> 2 weeks	35 (76.1)
Implementation of CW recommendation regarding MRI/arthroscopy, *n* (yes), %	
MRI, *n* (%)	42 (36.2)
Arthroscopy, *n* (%)	58 (50.0)

*n* = 116

^a^*n* = 46

^b^*n* = 60

^c^*n* = 92

^d^*n* = 103

^e^*n* = 56

^f^Pain measured on a visual analogue scale (VAS), 0 (no pain)—10 (unbearable pain)

**Table 2:** Background characteristics of orthopaedic surgeons, characteristics of care delivery and implementation of MRI/arthroscopy clinical guidelines (*n *= 252)

Background characteristics	
Age in years, (mean, SD)^a^	47.2 (8.5)
Female, *n* (%)	25 (9.9)
Years of work experience as orthopaedic surgeon (mean, SD)	12.0 (8.0)
Work region, *n* (%)	
North	85 (33.7)
Middle	105 (41.7)
South	62 (24.6)
New patients ≥ 50 years with knee complaints seen per month, *n* (%)	
0–1	1 (0.4)
2–5	9 (3.6)
6–10	12 (4.8)
11–20	34 (13.5)
> 20	196 (77.8)
Number of MRI scans ordered per month, *n* (%)	
0–1	70 (27.8)
2–5	81 (32.1)
6–10	55 (21.8)
11–20	35 (13.9)
> 20	11 (4.4)
Number of arthroscopies carried out per month, mean (SD)	
0–1	107 (42.5)
2–5	97 (38.5)
6–10	37 (14.7)
11–20	9 (3.6)
> 20	2 (0.8)
Percentage of patients ≥ 50 years undergoing an arthroscopy because of locking symptoms, *n* (%)	
0–10%	41 (16.3)
11–20%	11 (4.4)
21–30%	16 (6.3)
31–40%	11 (4.4)
41–50%	14 (5.6)
51–60%	17 (6.7)
61–70%	16 (6.3)
71–80%	38 (15.1)
81–90%	45 (17.9)
91–100%	43 (17.1)
Characteristics of care delivery	
Centre has its own MRI scan, *n* (%)^b^	228 (90.5)
Waiting time for MRI scan, *n* (%)	
≤ 2 weeks	125 (51.0)
> 2 weeks	120 (49.0)
Waiting time for arthroscopy, *n* (%)^b^	
≤ 2 weeks	60 (24.5)
> 2 weeks	185 (75.5)
Implementation of CW recommendation regarding MRI/arthroscopy, *n* (yes), %	
MRI, *n* (%)	203 (80.6)
Arthroscopy, *n* (%)	208 (82.5)

*n* = 252

^a^*n* = 244

^b^*n* = 245

### Factors influencing the use of MRI and arthroscopy among patients

Table 3 shows that most patients agreed with the statements “Good contact with physical therapist helped me to persevere the physical therapy treatments” (90%), “Good guidance of the physical therapist helped me to persevere all physical therapy treatments” (90%), “I have an additional coverage” (85%), and “Physical activity was difficult because of pain” (84%).

**Table 3:** Presence factors influencing the implementation of CW recommendation for MRI and/or arthroscopy reported by patients (*n *= 116)

	Agree *n* (%)
Individual professional	
Orthopaedic surgeon asked which treatments the patient previously received for his/her knee complaints	89 (76.7)
Orthopaedic surgeon listened well to patient’s wishes	89 (76.7)
Orthopaedic surgeon thought along with patient	86 (74.1)
Orthopaedic surgeon takes time to explain benefits and drawbacks of treatment options (medication, physical therapy or arthroscopy)	81 (69.8)
Orthopaedic surgeon explained the added value of MRI	60 (51.7)
Orthopaedic surgeon explained the benefits and drawbacks of an arthroscopy	60 (51.7)
Orthopaedic surgeon preferred an arthroscopy	47 (40.5)
Patient	
Physical activity was difficult because of pain	97 (83.6)
Patient searched for information before visiting the orthopaedic surgeon	73 (62.9)
Patient wanted an arthroscopy only if it was the last treatment option	55 (47.4)
Patient expected to undergo an MRI scan before the consult with the orthopaedic surgeon	37 (31.9)
Patient expected to undergo an arthroscopy prior to the consult with the orthopaedic surgeon	39 (33.6)
Patient preferred to undergo an MRI scan during the consult with the orthopaedic surgeon	54 (46.6)
Patient preferred to undergo an arthroscopy during the consult with the orthopaedic surgeon	52 (44.8)
Patient previously had negative experiences with physical therapy	15 (12.9)
In a situation in which different treatment options have approximately the same results:	
… patient prefers to decide about the treatment him/herself (active)	35 (30.2)
… patient prefers to decide about the treatment together with the orthopaedic surgeon (shared)	61 (52.6)
… patient prefers to let the orthopaedic surgeon decide about the treatment (passive)	20 (17.2)
In the situation of the consult of the patient with his/her orthopaedic surgeon:	
… patient decided about the treatment him/herself (active)	30 (25.9)
… patient decided about the treatment together with the orthopaedic surgeon (shared)	41 (35.3)
… patient let the orthopaedic surgeon decide about the treatment (passive)	45 (38.8)
Social context	
Good consultation between orthopaedic surgeon and physical therapist^a^	17 (29.3)
People in patient’s environment recommended an MRI scan	33 (28.4)
People in patient’s environment had good experiences with arthroscopy	48 (41.4)
People in patient’s environment stimulated to keep on moving despite pain	75 (64.7)
Organisational context	
Sufficient time for the orthopaedic surgeon to explain all treatment options (medication, physical therapy or arthroscopy), including benefits and drawbacks	80 (69.0)
Good contact with physical therapist helped patient to carry on with non-surgical therapy^b^	64 (90.1)
Good guidance of the physical therapist helped the patient withstand the duration of the non-surgical therapy^b^	64 (90.1)
Economic and political context	
Additional payment for physical therapy not (fully) covered by insurance	99 (85.3)
Patient preferred an arthroscopy because physical therapy was not covered by insurance	4 (3.4)

^a^Question answered by 58 of the 116 participants (*n* = 58)

^b^Question answered by 71 of the 116 participants (*n* = 71)

Table 4 shows that undergoing an MRI was associated with five barriers and two background characteristics among patients. Undergoing a knee arthroscopy was associated with five barriers, three facilitators and one background characteristic. From these, the orthopaedic surgeon’s explanation about the added value of an MRI [OR 0.18 (95% CI 0.07–0.47)] and the preference of the patient for an MRI [OR 0.27 (95% CI 0.08–0.92)] remained as independent factors associated with reduced implementation of the CW recommendation regarding MRI, whereas a higher age [OR 1.07 (95% CI 1.01–1.14)] was associated with higher implementation. For arthroscopy, the preference of the orthopaedic surgeon for arthroscopy [OR 0.03 (95% CI 0.00–0.22)] and positive experiences of people in the patient’s environment [OR 0.03 (95% CI 0.00–0.39)] remained as independent factors associated with reduced implementation of the CW recommendation regarding arthroscopy.

**Table 4:** Influencing factors, background characteristics and received care reported by patients for implementation of CW recommendations (*n *= 116) (univariate and multivariate analyses)

	Univariate analyses		Multivariate analyses	
	Implementation of CW MRI recommendation OR (95% CI)	Implementation of CW arthroscopy recommendation OR (95% CI)	Implementation of CW MRI recommendation OR (95% CI)	Implementation of CW arthroscopy recommendation OR (95% CI)
Factors influencing the implementation of the CW recommendations				
Individual professional				
Orthopaedic surgeon asked which treatments the patient previously received for his/her knee complaints	1.18 (0.48–2.92) (+)	0.61 (0.26–1.47) (−)	x	x
Orthopaedic surgeon listened well to patient’s wishes	0.95 (0.39–2.33) (−)	0.91 (0.38–2.15) (−)	x	x
Orthopaedic surgeon thought along with the patient	0.67 (0.29–1.56) (−)	1.00 (0.44–2.30)	x	x
Orthopaedic surgeon takes time to explain benefits and drawbacks of treatment options (medication, physical therapy, or arthroscopy)	x	0.92 (0.42–2.04) (−)	x	x
Orthopaedic surgeon explained the added value of an MRI	0.15 (0.06–0.36) (−)	x	**0.18 (0.07**–**0.47)** (−)	x
Orthopaedic surgeon explained the benefits and drawbacks of an arthroscopy	x	0.30 (0.14–0.64) (−)	x	0.61 (0.09–3.94) (−)
Orthopaedic surgeon preferred an arthroscopy	x	0.02 (0.01–0.06) (−)	x	**0.03 (0.00**–**0.22)** (−)
Patient				
Patient expected to undergo an MRI scan previous to the consult with the orthopaedic surgeon	0.45 (0.19–1.07) (−)	x	1.31 (0.35–4.90) (+)	x
Patient expected to undergo an arthroscopy previous to the consult with the orthopaedic surgeon	x	0.30 (0.13–0.68) (−)	x	4.88 (0.36–65.71) (+)
Patient preferred to undergo an MRI scan during the consult with the orthopaedic surgeon	0.21 (0.09–0.50) (−)	x	**0.27 (0.08**–**0.92)** (−)	x
Patient preferred to undergo an arthroscopy during the consult with the orthopaedic surgeon	x	0.12 (0.05–0.27) (−)	x	0.24 (0.04–1.65) (−)
Physical activity was difficult because of pain	1.28 (0.45–3.66) (+)	0.88 (0.33–2.36) (−)	x	x
Patient searched for information previous to the visit to the orthopaedic surgeon	0.42 (0.19–0.93) (−)	1.25 (0.59–2.66) (+)	0.84 (0.31–2.28) (−)	x
Patient wanted an arthroscopy only if it was the last treatment option	x	0.81 (0.39–1.69) (−)	x	x
Patient previously had negative experiences with physical therapy	0.60 (0.18–2.03) (−)	1.17 (0.39–3.46) (+)	x	x
In a situation in which different treatment options have approximately the same results…:				
… patient prefers to decide about the treatment him/herself	0.60 (0.19–1.91) (−)	1.78 (0.58–5.43) (+)	x	x
… patient prefers to decide about the treatment together with the orthopaedic surgeon	0.97 (0.35–2.73) (−)	1.55 (0.56–4.32) (+)	x	x
… patient prefers to let the orthopaedic surgeon decide about the treatment	Reference category	Reference category	x	x
In the situation of the consult of the patient with his/her orthopaedic surgeon:				
… patient decided about the treatment him/herself	0.91 (0.34–2.40) (−)	1.97 (0.77 –5.08) (+)	x	x
… patient decided about the treatment together with the orthopaedic surgeon	1.16 (0.48–2.78) (+)	0.89 (0.38–2.09) (−)	x	x
… patient let the orthopaedic surgeon decide about the treatment	Reference category	Reference category	x	x
Social context				
Good consultation between orthopaedic surgeon and physical therapist^a^	x	0.80 (0.26–2.48) (−)	x	x
People in patients’ environment recommended an MRI scan	0.37 (0.14–0.95) (−)	x	0.64 (0.19–2.12) (−)	x
People in patients’ environment had good experiences with arthroscopy	x	0.13 (0.06–0.31) (−)	x	**0.03 (0.00**–**0.39)** (−)
People in patients’ environment stimulated to keep on moving despite the pain	1.36 (0.61–3.04) (+)	1.99 (0.92–4.32) (+)	x	2.77 (0.24–31.44) (+)
Organisational context				
Sufficient time for the orthopaedic surgeon to explain all treatment options (medication, physical therapy or arthroscopy), including risks and benefits	x	1.18 (0.54–2.58) (+)	x	x
Good contact with physical therapist helped the patient to carry on with non-surgical therapy^b^ (–)	x	8.22 (0.94–72.33) (+)	x	7.69 (0.01–5090.47) (+)
Good guidance of the physical therapist helped the patient to withstand the duration of the non-surgical therapy^b^	x	8.22 (0.94–72.33) (+)	x	5.95 (0.01–3504.06) (+)
Economic and political context				
Additional payment for physical therapy (fully) covered by insurance	0.78 (0.27–2.23) (−)	1.15 (0.41–3.22) (+)	x	x
Patient preferred an arthroscopy because physical therapy was not covered by insurance	1.80 (0.24–13.27) (+)	1.00 (0.14–7.35)	x	x
Background characteristics				
Age	1.09 (1.03–1.15)) (+)	0.98 (0.94–1.03) (−)	**1.07 (1.01**–**1.14) (+)**	x
Gender	0.90 (0.41–1.94) (−)	1.94 (0.91–4.13) (+)	x	2.28 (0.31–16.82) (+)
Province of residence				
North	0.66 (0.28–1.55) (−)	0.94 (0.43–2.09) (−)	x	x
Middle	Reference category	Reference category	x	x
South	1.61 (0.43–6.12) (+)	0.63 (0.16–2.43) (−)	x	x
Level of education				
Basic	2.32 (0.50–10.69) (+)	2.69 (0.50–14.51) (+)	3.45 (0.57–20.88) (+)	x
Intermediate	0.50 (0.22–1.13) (−)	0.69 (0.32–1.47) (−)	0.66 (0.25–1.77) (−)	x
High	Reference category	Reference category	Constant factor	x
Pain before consult with orthopaedic surgeon	0.97 (0.81–1.15) (−)	0.94 (0.80–1.12) (−)	x	x
Diagnosis of orthopaedic surgeon was a locked knee^c^	2.63 (0.50–13.72) (+)	x	x	x
Received care				
Time between start of knee complaints and the consult with the general practitioner^d^	0.77 (0.32–1.84) (−)	0.91 (0.40–2.07) (−)	x	x
Time between consult with the general practitioner and consult with orthopaedic surgeon^e^	0.81 (0.30–2.19) (−)	0.88 (0.33–2.35) (−)	x	x

OR (95% CI) = odds ratio (95% confidence interval), (−) barrier, OR < 1, (+) facilitator, OR > 1. In bold: *p* values ≤ 0.05, *n* = 116

^a^*n* = 58

^b^*n* = 71

^c^*n* = 52

^d^*n* = 92

^e^*n* = 103

### Factors influencing the use of MRI and arthroscopy among orthopaedic surgeons

Table 5 shows that most orthopaedic surgeons agreed with the statements “asking questions about the previous non-surgical treatments” (98%), the familiarity with the CW recommendation for MRI (99%) and arthroscopy (98%) as influential factors for implementation.

**Table 5:** Orthopaedic surgeons’ agreement with factors influencing the implementation of the CW recommendation regarding MRI and/or arthroscopy (*n *= 252)

Level	Agree *n* (%)
Individual professional	
Orthopaedic surgeon asks about previously received non-surgical treatments (physical therapy, medication, nutritional advice when BMI > 25 and lifestyle advice)	248 (98.4)
Orthopaedic surgeon prescribes one or more non-surgical treatments (physical therapy, medication, nutritional advice when BMI > 25 and lifestyle advice) if patient did not receive all non-surgical treatment care yet	240 (95.2)
Belief in effectivity of non-surgical treatment strategy (physical therapy, medication, nutritional advice when BMI > 25 and lifestyle advice) for knee complaints of patients ≥ 50 years	234 (92.9)
Fully familiar with the CW recommendation for MRI	249 (98.8)
Agrees with the CW recommendation for MRI	228 (90.5)
Higher valuation of own experience with MRI than of existing evidence	90 (35.7)
Belief in value of MRI over fixed flexion view	109 (43.3)
Fully familiar with the CW recommendation for arthroscopy	248 (98.4)
Agrees with the CW recommendation for arthroscopy	234 (92.9)
Higher valuation of own experience with arthroscopy than of existing evidence	73 (29.0)
Belief in value of arthroscopy for patients ≥ 50 years with knee complaints, without ‘locked knee’ complaints, despite possible complications and risks	50 (19.8)
Important to perform arthroscopy as soon as possible for patients ≥ 50 years with knee complaints, without ‘locked knee’ complaints	5 (2.0)
Actively searches for latest knowledge about evidence and guidelines for diagnosis/treatment of knee complaints	199 (79.0)
Orthopaedic surgeon wants to meet patients’ expectations^a^	147 (59.5)
Orthopaedic surgeon is able to clarify to the patient whether an MRI scan is necessary, even if the patient has a contradictory opinion at first^a^	169 (68.4)
Orthopaedic surgeon is able to clarify to the patient whether an arthroscopy is necessary, even if the patient has a contradictory opinion at first^a^	188 (76.1)
Patient	
Orthopaedic surgeon notices that patients are well prepared for the consult by gaining knowledge	67 (26.6)
Patients’ level of knowledge is sufficient to make a shared decision about treatment	80 (31.7)
Patients ≥ 50 years with knee complaints have certain expectations about diagnostics and treatment when they come to the consult^a^	134 (94.7)
Most patients find it difficult that the CW recommendation for MRI also applies to them^a^	190 (76.9)
Most patients find it difficult that the CW recommendation for arthroscopy also applies to them^a^	170 (68.8)
Social context	
Colleagues all follow the CW recommendation for MRI and arthroscopy^b^	155 (63.3)
Colleagues tell me when I do not follow the guidelines^b^	197 (80.4)
Colleagues are in favour of non-surgical treatments (physical therapy, medication, nutritional advice and lifestyle advice) ^b^	220 (89.8)
Organisational context	
Able to make clear arrangements with primary care (GP, physical therapist, dietician)	188 (74.6)
Good feedback from primary care (GP, physical therapist, dietician) to orthopaedic surgeon about patient progress	139 (55.2)
Enough time to keep knowledge of guidelines up to date	156 (61.9)
Enough time to explain to the patient which diagnosis and treatment options are applicable to the patient’s situation^a^	164 (66.4)
Pressure of production MRI^b^	17 (6.9)
Pressure of production arthroscopy^b^	17 (6.9)
Economic and political context	
Financial reasons determine patient preference (arthroscopy more often covered by insurance than non-surgical treatment^a^)	84 (34.0)
Medicolegal substantiation to follow the CW recommendation for MRI^b^	27 (11.0)
Medicolegal substantiation to follow the CW recommendation for arthroscopy^b^	7 (2.9)

*n* = 252

^a^*n* = 247

^b^*n* = 245

Table 6 shows that implementation of the CW recommendation regarding MRI was associated with four barriers and six facilitators among orthopaedic surgeons in univariate analysis. Implementation of the CW recommendation regarding arthroscopy was associated with two barriers, five facilitators and three background characteristics. From these, agreement with the CW recommendation regarding MRI [OR 12.10 (95% CI 3.51–41.64)] remained as an independent factor associated with higher implementation of the CW recommendation in multivariate analysis, whereas higher valuation of own experience than existing evidence [OR 0.41 (95% CI 0.19–0.88)] and higher estimated patients’ knowledge to participate in shared decision-making [OR 0.38 (95% CI 0.17–0.88)] were associated with reduced implementation. Knowledge of [OR 58.17 (95% CI 2.63–1287.24)] and agreement with the CW recommendations regarding arthroscopy [OR 37.45 (95% 5.39–260.24)] as well as actively searching for newest evidence and guidelines [OR 3.28 (95% CI 1.19–9.08)] were associated with higher implementation of the CW recommendation regarding arthroscopy, whereas higher valuation of own experience than existing evidence [OR 0.17 (95% CI 0.07–0.46)] and belief in the value of arthroscopy [OR 0.28 (95% CI 0.10–0.81)] were associated with reduced implementation.

**Table 6:** Influencing factors and background characteristics reported by orthopaedic surgeons for the implementation of the CW recommendations (*n *= 252) (univariate and multivariate analyses)

	Univariate analyses		Multivariate analyses	
	Acts according to CW MRI recommendation OR (95% CI)	Acts according to arthroscopy CW recommendation OR (95% CI)	Acts according to CW MRI recommendation OR (95% CI)	Acts according to arthroscopy CW recommendation OR (95% CI)
Factors influencing the implementation of CW recommendations				
Individual professional				
Orthopaedic surgeon asks about previously received non-surgical treatments	1.39 (0.14–13.65) (+)	x^d^	x	x
Orthopaedic surgeon uses step-by-step treatment strategy	2.17 (0.63–7.51) (+)	2.50 (0.72–8.70) (+)	x	x
Belief in effectivity of non-surgical treatment strategy	2.91 (1.07–7.95) (+)	2.58 (0.91–7.29) (+)	0.96 (0.22–4.27) (−)	0.31 (0.03–3.10) (−)
Knowledge about the CW recommendation for MRI	2.09 (0.19–23.57) (+)	x	x	x
Agree with the CW recommendation for MRI	14.88 (5.72–38.70) (+)	x	**12.10 (3.51**–**41.64) (+)**	x
Higher valuation of own experience with MRI than of existing evidence	0.27 (0.14–0.51) (−)	x	**0.41 (0.19**–**0.88)** (−)	x
Belief in value of MRI over fixed flexion view	0.36 (0.19–0.69) (−)	x	0.49 (0.23–1.07) (−)	x
Orthopaedic surgeon actively searches for latest knowledge about evidence and guidelines for diagnosis/treatment of knee complaints	2.46 (1.24–4.91) (+)	2.64 (1.30–5.37) (+)	1.87 (0.79–4.45) (+)	**3.28 (1.19**–**9.08) (+)**
Knowledge about the CW recommendation for arthroscopy	x	15.15 (1.54–149.25) (+)	x	**58.17 (2.63**–**1287.24) (+)**
Agrees with the CW recommendation for arthroscopy	x	58.86 (12.85–269.66) (+)	x	**37.45 (5.39**–**260.24) (+)**
Higher valuation of own experience with arthroscopy than of existing evidence	x	0.14 (0.07–0.28) (−)	x	**0.17 (0.07**–**0.46)** (−)
Belief in value of arthroscopy despite possible complications and risks	x	0.10 (0.05–0.22) (−)	x	**0.28 (0.10**–**0.81)** (−)
Important to perform arthroscopy as soon as possible	x	0.84 (0.09–7.73) (−)	x	x
Orthopaedic surgeon wants to meet patients’ expectations^a^	0.80 (0.41–1.54) (−)	1.20 (0.62–2.34) (+)	x	x
Orthopaedic surgeon is able to clarify to the patient whether an MRI scan is necessary, even if the patient has a contradictory opinion at first^a^	1.29 (0.66–2.52) (+)	x	x	x
Orthopaedic surgeon is able to clarify to the patient whether an arthroscopy is necessary, even if the patient has a contradictory opinion at first^a^	x	1.29 (0.62–2.72) (+)	x	x
Patient				
Orthopaedic surgeon notices that patients are well prepared for the consult by gaining knowledge	0.62 (0.32–1.20) (−)	0.64 (0.32–1.30) (−)	x	x
Patients’ level of knowledge is sufficient to make a shared decision about treatment	0.55 (0.29–1.04) (−)	1.00 (0.50–2.00)	**0.38 (0.17**–**0.88)** (−)	x
Patients ≥ 50 years with knee complaints have certain expectations about diagnostics and treatment when they come to the consult^a^	0.76 (0.16–3.57) (−)	0.86 (0.18–4.01) (−)	x	x
Most patients find it difficult that the CW recommendation for MRI also applies to them^a^	0.26 (0.09–0.75) (−)	x	0.34 (0.10–1.16) (−)	x
Most patients find it difficult that the CW recommendation for arthroscopy also applies to them^a^	x	0.53 (0.24–1.17) (−)	x	x
Social context				
All colleagues follow the CW recommendation for MRI and arthroscopy^b^	2.09 (1.10–3.97) (+)	4.79 (2.37–9.69) (+)	1.54 (0.66–3.60) (+)	2.51 (0.94–6.70) (+)
Colleagues speak to me when I do not follow the guidelines^b^	1.78 (0.85–3.72) (+)	1.79 (0.84–3.81) (+)	x	x
Positive attitude of colleagues towards non-surgical treatments (physical therapy, medication, nutritional advice and lifestyle advice)^b^	3.30 (1.38–7.91) (+)	1.99 (0.77–5.11) (+)	1.13 (0.32–3.94) (+)	x
Organisational context				
Orthopaedic surgeon is able to make clear arrangements with primary care (GP, physical therapist, dietician)	1.22 (0.61–2.46) (+)	1.68 (0.83–3.38) (+)	x	x
Good feedback from primary care (GP, physical therapist, dietician) to orthopaedic surgeon about patient’s progress	1.00 (0.54–1.88)	1.60 (0.83–3.09) (+)	x	x
Enough time to keep knowledge of guidelines about diagnosis/treatment of knee complaints up to date	1.75 (0.93–3.28) (+)	0.81 (0.41–1.61) (−)	2.14 (0.95–4.84) (+)	x
Enough time to explain the patient which diagnosis and treatment options are applicable to the patients’ situation^a^	1.03 (0.52–2.00) (+)	0.95 (0.47–1.90) (−)	x	x
Pressure of production MRI^b^	1.84 (0.41–8.36) (+)	x	x	x
Pressure of production arthroscopy^b^	x	0.99 (0.27–3.62) (−)	x	x
Waiting time for MRI scan	1.00 (0.53–1.89)	x	x	x
Waiting time for arthroscopy	x	1.64 (0.80–3.36) (+)	x	x
Economic and political context				
Financial reasons determine patient preference because arthroscopy is more often covered by insurance than non-surgical treatment^a^	x	1.88 (0.88–4.03) (+)	x	x
Medicolegal substantiation to follow the CW recommendation for MRI^b^	0.64 (0.25–1.62) (−)	x	x	x
Medicolegal substantiation to follow the CW recommendation for arthroscopy^b^	x	0.52 (0.10–2.78) (−)	x	x
Centre has its own MRI scan	0.73 (0.44–1.20) (−)	x	x	x
Background characteristics				
Gender	0.96 (0.34–2.70) (−)	1.12 (0.37–3.45) (+)	x	x
Age^c^	1.00 (0.97–1.04)	0.96 (0.92–1.00) (−)	x	1.10 (0.88–1.36) (+)
Years of experience as an orthopaedic surgeon	1.00 (0.96–1.04)	0.95 (0.92–0.99) (−)	x	0.88 (0.70–1.11) (−)
Work setting				
University medical centre	2.20 (0.47–10.30) (+)	1.95 (0.42–9.19) (+)	x	x
Teaching hospital	1.61 (0.77–3.67) (+)	1.89 (0.85–4.18) (+)	x	x
Private clinic	0.63 (0.27–1.47) (−)	0.55 (0.23–1.31) (−)	x	x
General hospital	Reference category	Reference category	x	x
Work region				
North	Reference category	Reference category	x	Reference category
Middle	1.39 (0.69–2.82) (+)	2.54 (1.17–5.53) (+)	x	1.97 (0.61–6.37) (+)
South	1.60 (0.69–3.71) (+)	1.52 (0.67–3.44) (+)	x	0.98 (0.30–3.16) (−)

OR (95% CI) = odds ratio (95% confidence interval), (−) barrier, OR < 1, (+) facilitator, OR > 1. In bold: *p* values ≤ 0.05

*n* = 252

^a^*n* = 247

^b^*n* = 245

^c^*n* = 244

^d^Could not be estimated

## Discussion

That the implementation of CW recommendations to reduce unnecessary MRIs and knee arthroscopies was hampered by patient preferences for MRI, positive experiences with arthroscopies in the patient’s environment, orthopaedic surgeons’ preferences for arthroscopy and their beliefs in the added value as well as valuing their own clinical experience to be more important than existing evidence were the most important findings of this study. On the other hand, orthopaedic surgeons’ knowledge of and agreement with the CW recommendations, as well as a proactive attitude towards searching for new evidence and guidelines facilitate implementation. Furthermore, older age of patients increased implementation of CW recommendations regarding MRI.

Previous studies were limited in only presenting the clinician perspective and mentioned clinician beliefs in the effectiveness of arthroscopic surgery [24, 28], clinicians’ need to meet patient expectations [12], perverse financial incentives [24, 28], fragmented clinical decision pathways [24] and insurance coverage [32] as possible barriers for implementation of CW recommendations regarding MRI and arthroscopy in degenerative knee disease. Our study results confirm that clinician beliefs hamper implementation, but perverse financial incentives for clinicians/hospitals, fragmented clinical decision pathways, and insurance coverage were not identified as barriers. Possibly, this can be explained by a different health-care system in which the studies are performed. In this study only 7% of the orthopaedic surgeons felt pressure to perform MRIs and arthroscopies because of production agreements and 75% of the orthopaedic surgeons reported that they were able to make clear agreements with GPs, physical therapists and dieticians about care delivery (Table 5). Furthermore, in this study 85% of the patients have reported that they have additional coverage for physical therapy treatment (Table 1).

Previous studies also showed that clinicians felt CW recommendations were hard to accept for patients [46], were worried about malpractice claims and did not have enough time to discuss the risks and benefits of imaging with the patient [36]. Around 70% of the orthopaedic surgeons reported in this survey that they thought patients had difficulties in accepting the CW recommendations (Table 5), but these were not independently associated with implementation in multivariate regression analyses. In addition, fear of malpractice claims and lack of time to discuss risks and benefits of imaging with the patients were also not found to hamper implementation: less than 11% of the orthopaedic surgeons felt they needed to request an MRI or perform an arthroscopy for medicolegal substantiation (Table 5). Sixty-six percent of orthopaedic surgeons reported they had enough time to explain treatment options to patients (Table 5) and 69% of the patients felt that their orthopaedic surgeon spent sufficient time to explain treatment options including risks and benefits (Table 3). This underlines the importance of assessment of factors influencing the implementation of every CW recommendation for different countries and also to include both the clinician and the patient perspective.

That the implementation of CW recommendations can also be influenced by patients was shown by this study, in addition to other studies. While previous studies regarding the use of MRI and arthroscopies in degenerative knee disease mainly mentioned clinician-related barriers [24, 28], it was shown by this study that also patients’ preferences for MRIs and positive experiences of people in their environment with arthroscopies hampered implementation of the CW recommendations. This is an important finding for future initiatives to improve implementation of CW recommendations. These should include both patient- and orthopaedic surgeon-directed strategies.

Implications for clinical practice are that the use of unnecessary MRIs and knee arthroscopy for patients with degenerative knee disease can potentially be reduced by strategies tailored to the identified barriers for implementation of the CW recommendations [11]. This reduction is of great importance as MRIs and arthroscopies for patients with degenerative knees provide no benefit for the patient, waste resources and may even cause harm to the patient [17, 35].

Although this study identified important starting points for improving implementation of CW recommendations, there are also limitations. First, only three patients and three orthopaedic surgeons were interviewed for survey development. However, after the second interview with the orthopaedic surgeon, no new information was obtained so more interviews were not required. Besides, the interviews were only used to explore if other factors should be included in the survey than already found in the literature. The second limitation is the retrospective nature of this study and the use of self-reported questions. Both patients and orthopaedic surgeons were asked to report the characteristics of received care/care delivery and barriers/facilitators retrospectively, and the use of CW recommendation. Therefore, it is possible that some patients and orthopaedic surgeons were not able to fully recall their respective care trajectory and provided care. Third, patients were self-selected after seeing the advertisements in the newspapers or on the websites, which may have caused selection bias. However, it seems that the patients who completed the survey represented the target group well [5].

## Conclusions

The identified factors give important starting points for improving implementation of the CW recommendations regarding MRIs and arthroscopies in degenerative knee disease. It seems important to search for strategies to change clinician beliefs on the added value of arthroscopies and MRIs. Moreover, these strategies should focus on the importance of clinical experiences based on evidence. Furthermore, patient-directed strategies are needed to address patient ‘subjective’ preferences based on social feedback from environment and social media. These may add to underlying misbeliefs on the value of MRI and arthroscopies in degenerative knee disease.

## Appendix 1: Items survey patient

**Table:**

Background characteristics	
Age	In years
Gender	Male, female
Region of residence	North (Friesland, Groningen, Flevoland, Noord-Holland, Drenthe, and Overijssel), middle (Zuid-Holland, Utrecht, and Gelderland) and south (Noord-Brabant, Zeeland, and Limburg)
Education level	Basic education (no or only primary education), intermediate education (prevocational secondary education, senior secondary vocational training, senior secondary general education, pre-university education) or higher education (higher professional education or university (bachelor’s, master’s or PhD degree)
Start of disease symptoms	0–3 months, 3–6 months, 6–12 months, and > 1 year
Diagnosis of locking symptoms by orthopaedic surgeon if patient received arthroscopy	Yes, no
Pain before visiting an orthopaedic surgeon	Visual analogue scale (VAS)
Pain at the moment of the survey	Visual analogue scale (VAS)
Health insurance	Basic insurance or additional coverage^a^
Characteristics of the received care	
History of caregivers	General practitioner (GP), physical therapist, orthopaedic surgeon, dietitian, and/or other
Received care modalities	MRI, arthroscopy and/or physical therapy (yes/no)
Time between start of knee complaints and visiting the GP	< 1 week, 1–6 weeks, > 6 weeks, or no idea
Waiting time between GP and orthopaedic surgeon	1–2 weeks, 3–4 weeks, 5–6 weeks, more than 6 weeks, or no idea
Waiting time MRI	1–2 weeks, 3–4 weeks, 5–6 weeks, more than 6 weeks, or no idea, not applicable (NA)
Waiting time arthroscopy	Waiting time arthroscopy (1–2 weeks, 3–4 weeks, 5–6 weeks, more than 6 weeks, or no idea, NA)
Preferred and actual role of the patient in treatment decision-making process	Control Preference Scale (CPS) [17]

^a^In the Netherlands, applying for a basic insurance is compulsory. In addition, patients can choose for an additional coverage

## Appendix 2: Items survey orthopaedic surgeon

**Table:**

Background characteristics	
Age	
Gender	Male, female
Years of working experience	
Work setting	University medical centre, teaching hospital, general hospital, independent treatment centre
Work region	North (Friesland, Groningen, Flevoland, Noord-Holland, Drenthe, and Overijssel), middle (Zuid-Holland, Utrecht, and Gelderland), and south (Noord-Brabant, Zeeland, and Limburg)
Number of new patients per month	
Number of MRIs and arthroscopies per month	
Percentage of patients undergoing an arthroscopy with locking symptoms	
Characteristics of care delivery	
Availability of MRI scan in hospital	Yes, no
Waiting time MRI	0–1 week, 1–2 weeks, 3–4 weeks, 4–5 weeks, or more than 5 weeks
Waiting time arthroscopy	0–1 week, 1–2 weeks, 3–4 weeks, 4–5 weeks, or more than 5 weeks
Implementation of CW recommendation	4-point Likert scale, ranging from “totally agree” (coded 1) till “totally disagree” (coded 4)

## Abbreviations

MRI: Magnetic resonance imaging
CW: Choosing Wisely
